# Both IDO1 and TDO contribute to the malignancy of gliomas via the Kyn–AhR–AQP4 signaling pathway

**DOI:** 10.1038/s41392-019-0103-4

**Published:** 2020-02-21

**Authors:** Lisha Du, Zikang Xing, Bangbao Tao, Tianqi Li, Dan Yang, Weirui Li, Yuanting Zheng, Chunxiang Kuang, Qing Yang

**Affiliations:** 1 State Key Laboratory of Genetic Engineering, School of Life Sciences, Fudan University, Songhu Road 2005, Shanghai, 200438 China; 2 Department of Neurosurgery, Xinhua Hospital, Shanghai Jiaotong University, School of Medicine, Kongjiang Road 1665, Shanghai, 200092 China; 3 Department of Chemistry, Tongji University, Siping Road 1239, Shanghai, 200092 China; 4 Institute of Science and Technology for Brain-Inspired Intelligence, Fudan University, Handan Road 220, Shanghai, 200433 China

**Keywords:** CNS cancer, Diseases of the nervous system

## Abstract

Indoleamine 2,3-dioxygenase 1 (IDO1), indoleamine 2,3-dioxygenase 2 (IDO2), and tryptophan 2,3-dioxygenase (TDO) initiate the first step of the kynurenine pathway (KP), leading to the transformation of l-tryptophan (Trp) into l-kynurenine (Kyn) and other downstream metabolites. Kyn is known as an endogenous ligand of the aryl hydrocarbon receptor (AhR). Activation of AhR through TDO-derived Kyn is a novel mechanism to support tumor growth in gliomas. However, the role of IDO1 and IDO2 in this mechanism is still unknown. Herein, by using clinical samples, we found that the expression and activity of IDO1 and/or TDO (IDO1/TDO) rather than IDO2 were positively correlated with the pathologic grades of gliomas. The expression of IDO1/TDO rather than IDO2 was positively correlated with the Ki67 index and overall survival. The expression of IDO1/TDO was positively correlated with the expression of aquaporin 4 (AQP4), implying the potential involvement of IDO1/TDO in glioma cell motility. Mechanistically, we found that IDO1/TDO accounted for the release of Kyn, which activated AhR to promote cell motility via the Kyn–AhR–AQP4 signaling pathway in U87MG glioma cells. RY103, an IDO1/TDO dual inhibitor, could block the IDO1/TDO–Kyn–AhR–AQP4 signaling pathway and exert anti-glioma effects in GL261 orthotopic glioma mice. Together, our results showed that the IDO1/TDO–Kyn–AhR–AQP4 signaling pathway is a new mechanism underlying the malignancy of gliomas, and suggest that both IDO1 and TDO might be valuable therapeutic targets for gliomas.

## Introduction

Gliomas are the most common primary central nervous system tumors in the brain.^1^ Despite advances in surgical resection, irradiation, and chemotherapy, the fatality rate of patients with gliomas remains high, and the survival time is only ~15 months for newly diagnosed patients.^2^ The poor prognosis for gliomas is mainly attributed to their diffuse infiltrative growth pattern.^3^ Migration and invasion are indispensable processes for the diffuse infiltrative growth of gliomas, and they are under tight regulation by various factors.^4^ Thus, insight from the molecular mechanisms underlying the migration and invasion of gliomas may provide novel therapeutic targets for the treatment of gliomas.

Recent studies suggest that the kynurenine pathway (KP), the major route of l-tryptophan (Trp) catabolism leading to the production of l-kynurenine (Kyn), plays an important role in the pathogenesis of gliomas.^5–10^ The enzymes indoleamine 2,3-dioxygenase 1 (IDO1) [EC 1.13.11.52], indoleamine 2,3-dioxygenase 2 (IDO2) [EC 1.13.11], and tryptophan 2,3-dioxygenase (TDO) [EC 1.13.11.11] initiate KP’s first step by converting Trp into N-formylkynurenine, which is almost immediately converted into Kyn.^11^ IDO1 and TDO are involved in the suppression of antitumor immunity and are potential targets for immunotherapy.^12–14^ TDO has been thought to be the central Trp-degrading enzyme in human glioma cells. TDO-dependent production of Kyn in gliomas has been implied to be a novel mechanism for suppressing antitumor immunity, and supporting tumor growth and motility through activation of the aryl hydrocarbon receptor (AhR).^13^ As isozymes of TDO, IDO1 and IDO2 are of great interest to determine whether they are involved in the pathological process of gliomas and contribute to the release of Kyn and the activation of the AhR in gliomas.

Although TDO and Kyn have been demonstrated to affect the motility of glioma cells,^13^ the underlying mechanism remains unclear. Invasive migration of glioma cells requires water channels called aquaporins for the redistribution of water.^15^ Aquaporin 4 (AQP4), one of the aquaporins, is highly expressed in the most malignant gliomas and participates in maintaining the water and ion balance.^16,17^ The knockdown of AQP4 impaired cell migration and invasion and induced glioblastoma cell apoptosis.^18,19^ Hence, it is interesting to address whether the TDO–Kyn–AhR signaling pathway modulates AQP4, thus affecting the motility of glioma cells. In addition, the effect of IDO1 on the motility of glioma cells and the mechanism behind the effect needs further elucidation.

In this study, the expression profiles of IDO1, IDO2, and TDO in paraffin-embedded tissue sections and the total activity of IDO1, IDO2, and TDO in serum samples from glioma patients at different pathologic grades were investigated. The correlations between IDO1, IDO2, and TDO expression and overall survival or Ki67 proliferative index were analyzed. The relationships between the expression of IDO1 and/or TDO (IDO1/TDO) and AQP4 expression or brain edema were evaluated. To elucidate the mechanism of the association between IDO1/TDO and the malignancy of gliomas, the effects of IDO1/TDO on AQP4 expression, cell migration and invasion, and cell morphology were studied in the U87MG glioma cell line. The contribution of IDO1/TDO to the production of Kyn, the effect of Kyn on AhR expression, and the potential binding of AhR to the AQP4 promoter were explored. Furthermore, the therapeutic efficacy of a dual inhibitor of IDO1/TDO in GL261 orthotopic glioma mice was examined. Our study provides new evidence that supports the contribution of IDO1 in addition to TDO to glioma malignancy, highlighting the therapeutic potential of IDO1 and TDO inhibitors in the treatment of gliomas.

## Results

### The expression and activity of IDO1/TDO were positively correlated with the pathologic grades of glioma

**Fig. 1:**
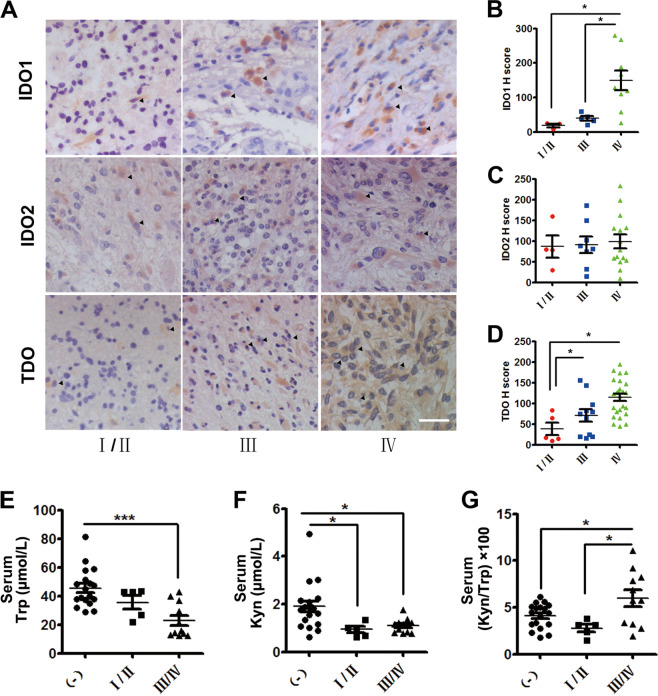
The expression and activity of IDO1/TDO were positively correlated with the pathologic grades of glioma. **a** Immunohistochemistry analysis of the expression of IDO1, IDO2, and TDO in gliomas of different pathologic grades (WHO grades I–IV. Upper panel: IDO1. Middle panel: IDO2. Lower panel: TDO. IDO1, IDO2, and TDO-positive cells (arrows). Magnification, ×200; scale bar, 100 μm). **b** Plot of IDO1 expression in gliomas of different pathologic grades (WHO grades I–IV: grade I/II, *n* = 3; grade III, *n* = 5; grade IV, *n* = 9). **c** Plot of IDO2 expression in gliomas of different pathologic grades (WHO grades I–IV: grade I/II, *n* = 4; grade III, *n* = 8; grade IV, *n* = 15). **d** Plot of TDO expression in gliomas of different pathologic grades (WHO grades I–IV: grade I/II, *n* = 5; grade III, *n* = 11; grade IV, *n* = 25). **e**–**g** HPLC analysis of Trp and Kyn levels and the Kyn/Trp ratio in the serum of 16 patients with glioma (grade I/II, *n* = 5; grade III/IV, *n* = 11) and 18 non-glioma patients. (−), non-glioma patients. I/II, WHO grades I and II; III/IV, WHO grades III and IV. Statistical significance was determined by one-way ANOVA followed by Dunnett’s test; data are presented as the mean ± SEM. *p < 0.05; ***p < 0.001.

Immunohistochemistry staining was used to measure the expression levels of IDO1, IDO2, and TDO in paraffin-embedded tissue sections of 75 glioma tissue donors (Supplementary Tables S1–S3). IDO1, IDO2, and TDO were expressed in a subgroup of patients (Fig. 1a, Table 1). In IDO1-, IDO2-, and TDO-expressing glioma tissue samples, it was found that the expression of IDO1 and TDO (Fig. 1a, b, d) rather than IDO2 (Fig. 1a, c) was positively correlated with the pathologic grades. Furthermore, in the serums of 34 blood donors (Supplementary Table S4), the total activity of IDO1, IDO2, and TDO in Trp catabolism was evaluated by measuring the concentration of Trp and Kyn using high-performance liquid chromatography (HPLC) and calculating the Kyn/Trp ratio. It was shown that the concentration of Trp in the high-grade (grade III/IV, *n* = 11) group was markedly lower than that in the low-grade (grade I/II, *n* = 5) group and non-glioma patient group (Fig. 1e). The concentration of Kyn in both the low- and the high-grade group was significantly lower than that in the non-glioma patient group (Fig. 1f). The ratio of Kyn/Trp in the high-grade group was much higher than that in the low-grade group and non-glioma patient group (Fig. 1g). Given that IDO2 expression was not correlated with the pathologic grades of glioma, it was deduced that the expression and activity of IDO1/TDO increased with the pathologic grades of glioma, and both IDO1 and TDO were involved in the malignancy of glioma.

**Table 1:** The number of glioma patients with positive expression of IDO1, IDO2, and TDO.

Group	Grade I	Grade II	Grade III	Grade IV	Total
IDO1 +	1	2	5	9	17
IDO2 +	0	4	8	15	27
TDO +	0	5	11	25	41

*IDO1* *+* IDO1 positive, *IDO2+* IDO2 positive, *TDO+* TDO positive

### The expression of IDO1/TDO was associated with poor prognosis in patients with glioma

**Fig. 2:**
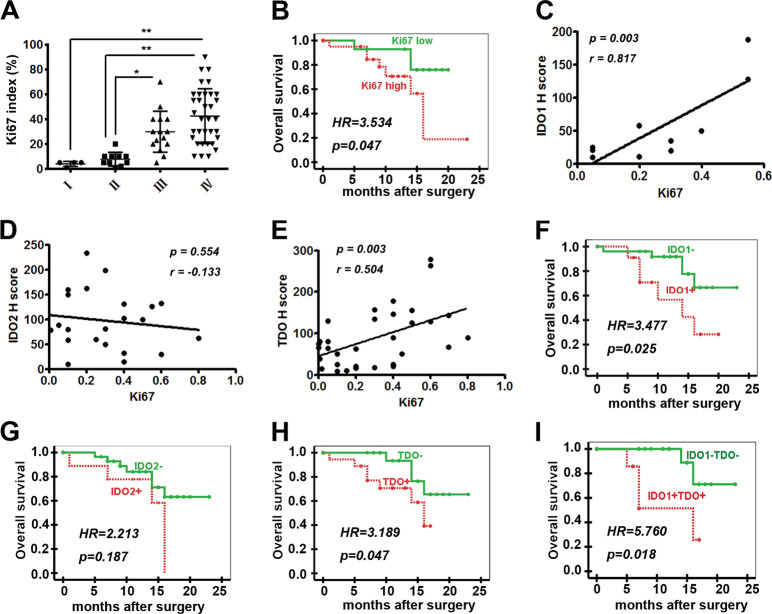
The expression of IDO1/TDO was negatively correlated with the prognosis of patients with glioma. **a** Ki67 index in gliomas of different pathologic grades (I: 4, II: 10, III: 15, and IV: 34). **b** Correlation between overall survival and the Ki67 index (Ki67 low, *n* = 17; Ki67 high, *n* = 23). **c–e** Correlation between the Ki67 index and H scores of IDO1 (*n* = 10), IDO2 (*n* = 22), and TDO (*n* = 34). **f**–**i** Correlation between overall survival and the expression of IDO1, IDO2, and TDO (IDO1 + , *n* = 13; IDO1−, *n* = 30; IDO2 + , *n* = 12; IDO2−, *n* = 31; TDO + , *n* = 23; TDO−, *n* = 20; IDO1 + TDO + , *n* = 9; IDO1–TDO−, *n* = 16). Statistical significance was determined by one-way ANOVA followed by Dunnett’s test; data are presented as the mean ± SEM (**a**). Kaplan–Meier curves of overall survival of patients with glioma were determined by log-rank test (**b**, **f**–**i**). Survival time is the interval between diagnosis and tumor-specific death, progression, or the last follow-up. *p < 0.05; **p < 0.01.

The Ki67 index and survival data of some patients with glioma were obtained (Supplementary Tables S1, S2). It is well known that an increased Ki67 index is associated with a higher grade of astrocytomas.^20^ Nevertheless, to date, studies of the independent prognostic value of the Ki67 index in glioma have revealed conflicting results.^21^ Herein, the correlation between the Ki67 index and the pathologic grades or overall survival of patients with glioma were analyzed. It was found that the Ki67 index increased with the pathologic grades (I–IV), and a high Ki67 index was associated with worse overall survival (Fig. 2a, b, Table 2). Furthermore, the protein expression levels of IDO1 and TDO were found to be positively correlated with the Ki67 index, whereas those of IDO2 were not (Fig. 2c–e).

**Table 2:** The correlations between IDO1, IDO2, TDO, and Ki67 expression and survival of glioma patients.

Variable	Total number of patient events	Death		Survival (month)		
		Number	%	Mean	95% CI	*P*
IDO1+	13	6	46.15	13.18	9.60–16.76	0.025
IDO1−	30	5	16.67	19.46	16.78–22.15	
IDO2+	12	4	33.33	12.94	9.11–16.78	0.187
IDO2−	31	7	22.58	18.88	16.34–21.41	
TDO+	23	7	30.43	12.95	10.44–15.46	0.047
TDO−	20	4	20.00	20.00	17.53–22.47	
IDO1+TDO+	9	4	44.44	11.60	7.56–15.74	0.018
IDO1–TDO−	16	2	12.50	20.75	18.07–23.44	
Ki67 high (≥mean)	23	8	34.78	14.44	15.65–20.18	0.047
Ki67 low (<mean)	17	3	17.65	17.92	11.13–17.76	

The potential correlation between the expression of IDO1, IDO2, and TDO and overall survival was further analyzed as shown in Table 2. It was found that the patients positive for IDO1 or TDO expression or IDO1 and TDO coexpression had lower overall survival (Fig. 2f, h, i). However, IDO2 expression was not correlated with the overall survival of glioma patients (Fig. 2g). Therefore, IDO1 or TDO expression or IDO1 and TDO coexpression could be used as independent prognostic factors for the overall survival of glioma patients. The results indicated that IDO1/TDO expression was negatively correlated with the prognosis of patients with gliomas.

### The expression of IDO1/TDO was positively correlated with AQP4 expression in patients with glioma

**Fig. 3:**
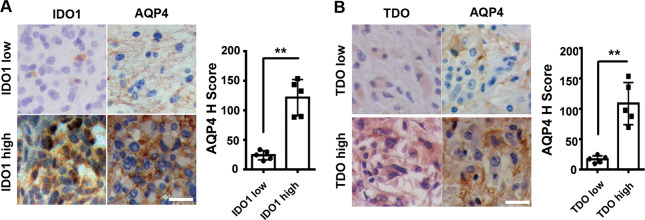
The expression of IDO1/TDO was positively correlated with AQP4 expression in glioma patients. **a** Immunohistochemistry analysis of the expression of AQP4 in the IDO1-low group (*n* = 5) and the IDO1-high group (*n* = 5). **b** Expression of AQP4 was immunohistochemically detected in the TDO-low group (*n* = 5) and TDO-high group (*n* = 5). Representative images and the accompanying statistical plots are presented (magnification, ×400 ; scale bar, 50 μm). Statistical significance was determined by Student's *t* test. **p < 0.01.

AQP4 plays an important role in proliferation, invasiveness, migration, and apoptosis in glioma cells.^22^ To explore the potential relationship between IDO1/TDO and glioma migration and invasion, paraffin-embedded tissue sections with IDO1 or TDO-positive expression were divided into four groups of IDO1-low group, IDO1-high group, TDO-low group, and TDO-high group according to the expression levels of IDO1 and TDO (for definition standards, see the Materials and methods section), and the expression level of AQP4 in these four groups was examined. Detailed information on grouping is shown in Supplementary Table S3, and five samples from each group were studied. It was revealed that the IDO1 or TDO-high group had significantly higher levels of AQP4 expression compared with the IDO1 or TDO-low group (Fig. 3a, b).

AQP4 upregulation has been reported to be associated with brain edema formation in malignant gliomas.^23^ Hence, it is worth clarifying the relationship between IDO1/TDO expression and the occurrence rate of brain edema. The incidence rates of brain edema in the IDO1−, IDO1 + , TDO−, and TDO + (for definition standards see the Materials and methods section) groups were 37%, 62%, 35%, and 52%, respectively (Supplementary Fig. S1, Supplementary Table S5). The IDO1 + group had a higher incidence rate of brain edema than the IDO1− group, although this difference was not statistically significant. A similar tendency was observed for the TDO + group and the TDO− group (Supplementary Fig. S1). Peritumoral edema is believed to loosen glioma tissue, facilitating tumor cell invasion.^24^ Thus, our results indicated that both IDO1 and TDO might be involved in glioma migration and invasion.

### Both IDO1 and TDO mediated the migration and invasion of glioma cells via Kyn

**Fig. 4:**
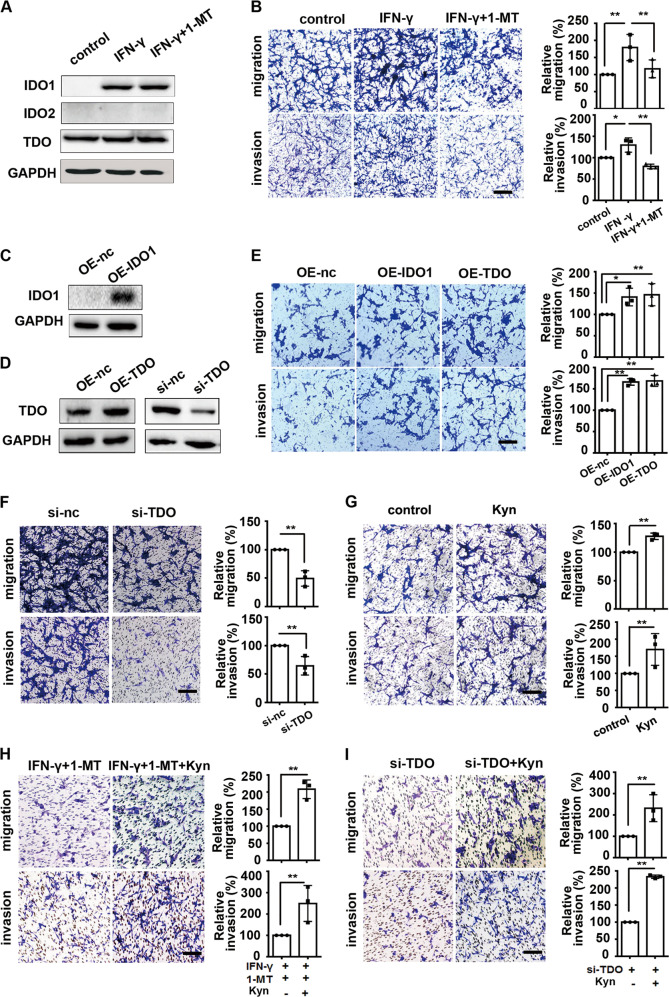
Both IDO1 and TDO mediated the migration and invasion of glioma cells via Kyn. **a** Western blot analysis of the effects of IFN-γ or IFN-γ + 1-MT on the expression of IDO1, IDO2, and TDO in U87MG cells. **b** Migration and invasion assays of U87MG cells treated with IFN-γ or IFN-γ + 1-MT. **c** Western blot analysis of the expression of IDO1 in IDO1-overexpressing U87MG cells (OE-IDO1). **d** Western blot analysis of the expression of TDO in TDO-overexpressing (OE-TDO) or TDO-knockdown (si-TDO) U87MG cells. **e** Migration and invasion assays of IDO1- or TDO-overexpressing U87MG cells. **f** Migration and invasion assays of TDO-knockdown U87MG cells. **g** Migration and invasion assays of U87MG cells treated with Kyn. **h** Migration and invasion assays of U87MG cells treated with IFN-γ + 1-MT or IFN-γ + 1-MT + Kyn. **i** Migration and invasion assays of si-TDO- or si-TDO + Kyn-treated U87MG cells. The designation of the different treatments is described in the Materials and methods section. Representative images and accompanying statistical plots are presented (magnification, ×200; scale bar, 100 μm). *n* = 3 per group. Statistical significance was determined by one-way ANOVA followed by Dunnett’s test (**b**, **e**) and Student's *t* test (**f**–**i**). Data are presented as the mean ± SEM. *p < 0.05; **p < 0.01.

The expression of IDO1/TDO was positively correlated with the expression of AQP4, which was associated with migration and invasion in glioma. We wanted to use human glioma cells to examine the effects of IDO1/TDO on cell migration and invasion. First, we investigated the expression of IDO1, IDO2, and TDO in different human glioma cell lines. We found that A172 cells were not suitable for studying IDO1/TDO because A172 cells endogenously expressed all three IDO1, IDO2, and TDO. U87MG and U251 cells endogenously expressed TDO, but lacked IDO1 and IDO2. However, IDO1 expression could be induced by IFN-γ. This result also showed that the expression of TDO and IDO2 was not affected by IFN-γ (Supplementary Fig. S2). The expression pattern of IDO1 and its isozymes in U87MG and U251 cells was similar, and the U87MG glioma cell line was chosen for further study. 1-Methyl-l-tryptophan (1-MT), one of the most commonly used IDO1 inhibitors, did not reverse the IFN-γ-induced IDO1 expression (Fig. 4a). Accordingly, treatment with IFN-γ enhanced cell migration and invasion, and supplementation with 1-MT reversed the effect on cell motility (Fig. 4b). Furthermore, the effect of IDO1/TDO overexpression on cell invasion and migration was examined. Compared with that in the control group (U87MG cells transfected with pcDNA3.1( + ) empty vector), the expression of IDO1/TDO as well as the migration and invasion ability were increased in both IDO1/TDO-overexpressing U87MG cells (Fig. 4c–e). Then, the effect of TDO knockdown by RNA interference on cell migration and invasion was examined. Compared with that of the si-nc group, the migration and invasion ability of TDO-knockdown U87MG cells was reduced (Fig. 4d, f). These results confirmed that both IDO1 and TDO mediated the migration and invasion of glioma cells (Fig. 4b, e, f). In addition, it was revealed that Kyn treatment promoted U87MG cell migration and invasion (Fig. 4g), and the decrease in glioma cell motility caused by IDO1 inhibition or TDO knockdown was restored when exogenous Kyn was supplemented (Fig. 4h, i). These results indicated that both IDO1 and TDO mediated U87MG cell migration and invasion via Kyn.

### Both IDO1 and TDO contributed to the production of Kyn, which upregulated AhR expression in glioma cells

**Fig. 5:**
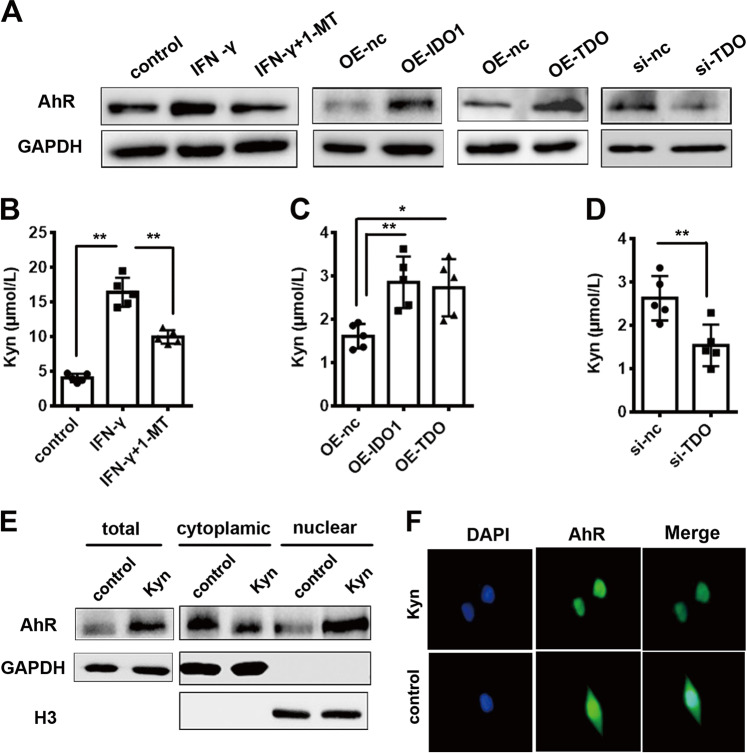
Both IDO1 and TDO contributed to the production of Kyn, which upregulated the expression of AhR in U87MG cells. **a** Western blot analysis of the expression of AhR in U87MG cells upon different treatments. **b** HPLC analysis of Kyn concentration in the media of U87MG cells stimulated with IFN-γ or IFN-γ + 1-MT. **c** HPLC analysis of Kyn concentration in the media of IDO1- or TDO-overexpressing U87MG cells. **d** HPLC analysis of Kyn concentration in the media of TDO-knockdown U87MG cells. **e** Western blot analysis of the expression of AhR in total, cytoplasmic, and nuclear protein samples of U87MG cells treated with Kyn. Cytoplasmic GAPDH and nuclear H3 were used as controls for normalization. **f** The nuclear localization of AhR (green) after Kyn treatment was determined by immunofluorescence staining. Nuclei were counterstained with DAPI (blue). The designation of the different treatments is described in the Materials and methods section. *n* = 5 per group. Statistical significance was determined by one-way ANOVA followed by Dunnett’s test (**b**, **c**) and Student's *t* test (**d**). Data are presented as the mean ± SEM. *p < 0.05; **p < 0.01.

TDO is thought to be the central enzyme responsible for producing Kyn, which activates AhR in glioma.^13^ Here, we examined whether IDO1, like TDO, also contributes to the production of Kyn, which activates AhR. In IDO1-overexpressing U87MG cells (induced by IFN-γ or transfected with pcDNA3.1( + )−IDO1), the expression of AhR was significantly increased. Furthermore, the expression of AhR was decreased when the activity of IDO1 was inhibited by 1-MT. Similarly, the expression of AhR was markedly increased in TDO-overexpressing U87MG cells (transfected with pcDNA3.1( + )−TDO). In addition, knockdown of TDO led to a decrease in AhR expression (Fig. 5a). Overexpression of IDO1 or TDO could promote Kyn release, whereas pharmacological inhibition of IDO1 or knockdown of TDO blocked Kyn release in U87MG cells (Fig. 5b–d), suggesting that IDO1 and TDO might upregulate AhR expression via Kyn. To confirm this, U87MG cells were treated with Kyn, and it was found that the total AhR protein level was increased. This result was similar to the report by Opitz et al. that AhR mRNA expression can be increased by Kyn treatment in the LN-18 and LN-308 glioma cell lines.^13^ Furthermore, decreased cytoplasmic localization paralleled by increased nuclear accumulation of AhR in comparison with the control were observed (Fig. 5e). Immunofluorescence staining showed that AhR was expressed in both the cytoplasm and nucleus without Kyn treatment, while supplementation with Kyn led to AhR translocation into the nucleus (Fig. 5f). The results of immunofluorescence staining showed a correlation between IDO1/TDO, Kyn, and AhR (Supplementary Fig. S3), which was consistent with the western blot results (Fig. 5a). All the data indicated that both IDO1 and TDO contributed to the production of Kyn, which upregulated AhR expression in glioma cells.

### The IDO1/TDO–Kyn–AhR signaling pathway modulated glioma cell migration and invasion via AQP4

**Fig. 6:**
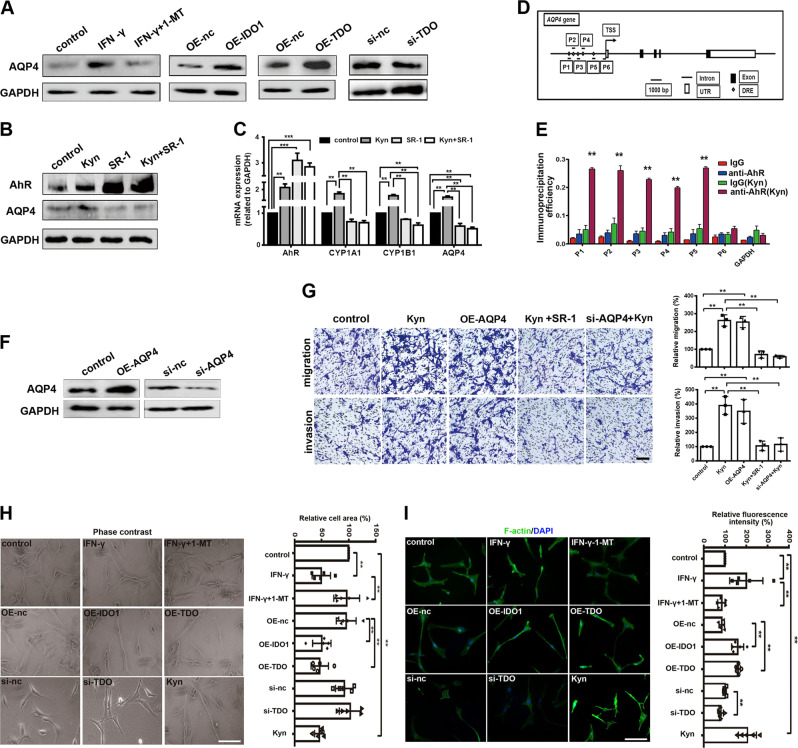
The IDO1/TDO–Kyn–AhR signaling pathway modulated glioma cell migration and invasion via AQP4. **a** Western blot analysis of the effects of IDO1 and TDO on AQP4 expression in U87MG cells. *n* = 3 per group. **b** Western blot analysis of the effects of Kyn, SR-1 (AhR inhibitor), or Kyn + SR-1 on the expression of AhR and AQP4 in U87MG cells. *n* = 3 per group. **c** qPCR analysis of the effects of Kyn, SR-1, or Kyn + SR-1 on the mRNA expression of AhR, CYP1A1, CYP1B1, and AQP4 in U87MG cells. *n* = 3 per group. **d** Schematic diagram of predicted AhR-binding sites (DREs) in the AQP4 promoter. **e** ChIP analysis of AhR binding to the AQP4 promoter in U87MG cells treated with Kyn. ChIP assays were performed with control IgG or anti-AhR antibody. Immunoprecipitated DNA was examined using qPCR and primers specific for the AQP4 promoter. Samples were normalized to the amount of input DNA. *n* = 3 per group. **f** Western blot analysis of the expression of AQP4 in AQP4-overexpressing (OE-AQP4) or knockdown (si-AQP4) U87MG cells. *n* = 3 per group. **g** Migration and invasion assays of U87MG cells under different conditions (magnification, ×200; scale bar, 100 μm). *n* = 3 per group. **h** Morphologic changes in U87MG cells upon different treatments were recorded by phase-contrast microscopy (left). Cell area per cell was quantified (right) (magnification, ×400; scale bar, 50 μm). *n* = 5 per group. **i** Phalloidin-iFluor 488-labeled F-actin (green) in U87MG cells upon different treatments was recorded by fluorescence microscopy. DAPI (blue) was used for nuclear staining (left). Fluorescence intensity of F-actin per cell was quantified (right). (Magnification, ×400; scale bar, 50 μm) *n* = 5 per group. The designation of the different treatments is described in the Materials and methods section. Statistical significance was determined by one-way ANOVA followed by Dunnett’s test. Data are presented as the mean ± SEM. **p < 0.01; ***p < 0.001.

The preceding results showed that the expression of IDO1 and TDO was positively related to the expression of AQP4, while Kyn produced by IDO1 and TDO upregulated the expression of AhR, so we examined whether and how the identified IDO1/TDO–Kyn–AhR signaling pathway regulated AQP4 expression. In IDO1-overexpressing U87MG cells (induced by IFN-γ or transfected with pcDNA3.1( + )–IDO1), the expression of AQP4 was significantly increased, while the expression of AQP4 was decreased when the activity of IDO1 was inhibited by 1-MT. Similarly, the expression of AQP4 was markedly increased in U87MG cells transfected with pcDNA3.1( + )–TDO, while the knockdown of TDO led to a decrease in the expression of AQP4 (Fig. 6a). In addition, AQP4 expression was increased upon Kyn treatment, and decreased when AhR activity was inhibited (Fig. 6b). Similar to the results in Fig. 5e, the expression of AhR in U87MG cells was increased when the cells were treated with Kyn (Fig. 6b). Unexpectedly, the expression of AhR was increased when the U87MG cells were treated with StemRegenin 1 (SR-1), an AhR inhibitor, or the combination of Kyn and SR-1 (Fig. 6b). The observed upregulation of expression might be due to compensation for the inhibited activity. The changes in the mRNA expression of AhR and AQP4 (Fig. 6c) were similar to the result of protein expression in Fig. 6b. The mRNA expression levels of cytochrome P450 1A1 (CYP1A1) and cytochrome P450 1B1 (CYP1B1), two AhR target genes, were increased upon Kyn treatment and decreased when the activity of AhR was inhibited (Fig. 6c). This result also confirmed that SR-1 inhibited AhR activity efficiently. The results of immunofluorescence staining (Supplementary Fig. S4) showed a correlation between IDO1/TDO, Kyn, and AQP4, which was consistent with the western blot results (Fig. 6a, b). It can be concluded that the IDO1/TDO–Kyn–AhR signaling pathway regulated AQP4 expression in glioma cells. In the nucleus, AhR and the AhR nuclear translocator form a heterodimer that interacts with the dioxin-responsive elements (DREs) located in the regulatory region of AhR target genes, resulting in their activation or repression.^25^ Bioinformatics analysis revealed the presence of five DRE sites in the AQP4 promoter (Fig. 6d). Chromatin immunoprecipitation (ChIP) experiments were performed to test whether AhR could bind to the DRE sites of the AQP4 promoter. It was found that Kyn supplementation caused a significant increase in immunoprecipitation efficiency (AhR binding to the DRE sites) in P1–P5, but not P6. GAPDH was used as a negative control gene. Without Kyn treatment, the immunoprecipitation efficiency was quite low at each site of P1–P5 (Fig. 6e). These results indicated that IDO1- and TDO-derived Kyn activated the binding of AhR to the AQP4 promoter and upregulated the expression of AQP4. Furthermore, the effect of overexpression due to transfection with the pENTER-AQP4 plasmid or knockdown due to transfection with siRNA against AQP4 was examined in U87MG cells (Fig. 6f). The motility of glioma U87MG cells was increased when AQP4 was overexpressed or Kyn was supplemented, while Kyn supplementation could not restore the cell motility decreased by AhR inhibition and AQP4 knockdown (Fig. 6g).

Furthermore, the effects of IDO1/TDO and Kyn on cell morphology, including cell area and the cytoskeleton, were investigated.^26^ Filament actin (F-actin) is an important component of the cytoskeleton, and the dynamic remodeling of F-actin provides the impetus for cell invasion and migration.^27^ It was found that IFN-γ treatment decreased the cell area, and the change was restored by supplementation with 1-MT (Fig. 6h). In addition, the overexpression of IDO1 and TDO as well as Kyn treatment caused a decrease in cell area, while the knockdown of TDO did not significantly change the cell area (Fig. 6h). As shown in Fig. 6i, F-actin expression was increased significantly after IFN-γ stimulation, which was almost eliminated by treatment with the IDO1 inhibitor 1-MT. Overexpression of IDO1 and TDO as well as Kyn treatment increased the expression of F-actin, and knockdown of TDO decreased the expression of F-actin (Fig. 6i). In summary, the IDO1/TDO–Kyn–AhR–AQP4 signaling pathway plays an important role in glioma cell migration and invasion.

### IDO1/TDO dual inhibitor RY103 inhibited the malignancy of glioma in vivo

RY103, a new dual inhibitor of IDO1/TDO, was found to be able to penetrate the blood–brain barrier in mice (Supplementary Fig. S5). The Trp and Kyn levels in the serums of GL261 orthotopic glioma mice and healthy mice were assessed using HPLC, and Kyn/Trp ratios were calculated. It was shown that the serum Trp levels in GL261 orthotopic glioma mice were similar to those in healthy mice. The serum Kyn levels and Kyn/Trp ratios in GL261 orthotopic glioma mice were higher compared with those in healthy mice (Supplementary Fig. S6A–C). Thus, the total activity of IDO1 and TDO was increased in GL261 orthotopic glioma mice. Tumor formation in GL261 orthotopic glioma mice was confirmed by hematoxylin and eosin staining, which clearly distinguished the tumor and nontumor areas (Supplementary Fig. S6D). The expression levels of IDO1, TDO, AhR, and AQP4 in the tumor areas of GL261 orthotopic glioma mice were higher than those in the corresponding brain tissue from healthy mice (Supplementary Fig. S6E). Our study showed that the GL261 orthotopic glioma mouse model was applicable for the pharmacodynamic study of IDO1/TDO inhibitors.

**Fig. 7:**
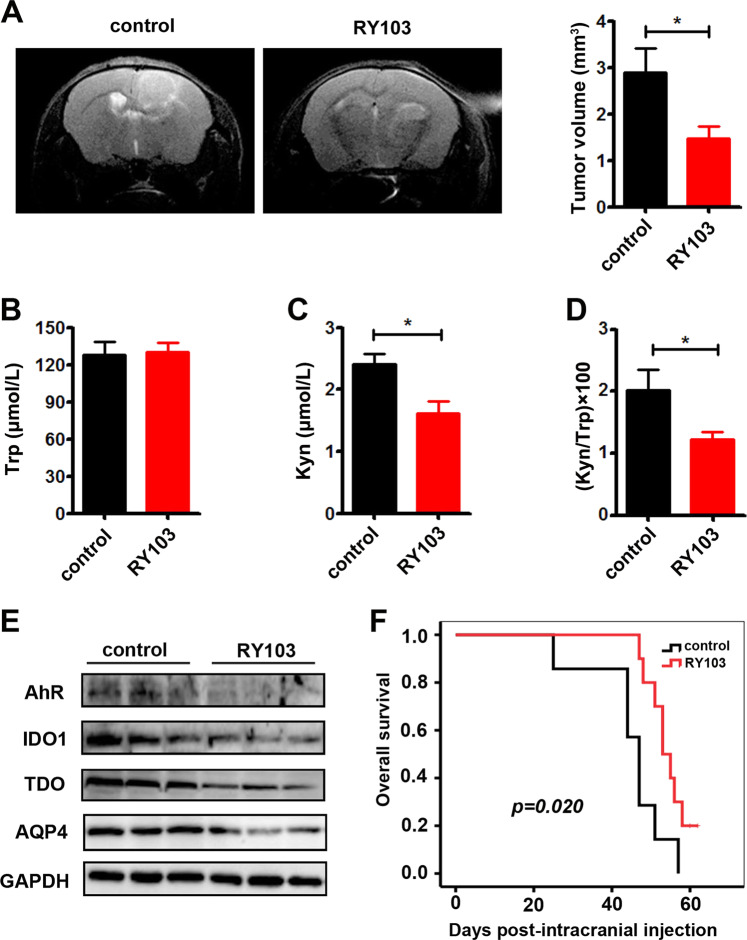
The IDO1/TDO dual inhibitor RY103 inhibited the malignancy of glioma in vivo. **a** The tumor volumes were assessed by MRI. Representative images (left) and quantification of tumor volume (right) are shown (control group, *n* = 4; RY103 group, *n* = 4). **b**–**d** HPLC analysis of the Trp and Kyn levels and Kyn/Trp ratios in the two groups (control group, *n* = 7; RY103 group, *n* = 7). **e** Western blot analysis of the expression of IDO1, TDO, AhR, and AQP4 in the tumor area (control group, *n* = 3; RY103 group, *n* = 3). The results were evaluated on the 21st day after therapy (**a**–**e**). **f** The survival curve of the two groups (control group, *n* = 7; RY103 group, *n* = 10). Statistical significance was determined by Student's *t* test (**a**–**d**). Kaplan–Meier curves of overall survival of glioma mice were determined by log-rank test (**f**). Data are presented as the mean ± SEM. *p < 0.05.

Then, the therapeutic efficacy of the IDO1/TDO dual inhibitor RY103 was examined in GL261 orthotopic glioma mice. The tumor volume in the RY103 group was markedly reduced compared with that in the control group, and representative magnetic resonance imaging (MRI) images are shown (Fig. 7a). It was shown that the serum Trp level in the RY103 group was similar to that in the control group, while the Kyn level and Kyn/Trp ratio were reduced in the RY103 group compared with the control group (Fig. 7b–d). Thus, the total activity of IDO1 and TDO was inhibited by the IDO1/TDO dual inhibitor RY103 in GL261 orthotopic glioma mice. Moreover, the expression levels of IDO1, TDO, AhR, and AQP4 in the RY103 group were lower than those in the control group (Fig. 7e). In addition, RY103 significantly prolonged the survival time of mice (Fig. 7f). Previous studies showed that the IDO1 inhibitor 1-MT (L or D stereoisomer) could not prolong GL261 orthotopic glioma mouse survival time.^28–30^ TDO inhibitors have not been tested in glioma animals. Our in vivo study confirmed that inhibition of IDO1/TDO could suppress the malignancy of glioma by blocking the IDO1/TDO–Kyn–AhR–AQP4 signaling pathway.

## Discussion

Although the aberrant activation of KP and expression patterns of the first rate-limiting KP enzyme in gliomas have been previously reported,^5,9,10,13,31–34^ the systemic and comprehensive studies of the expression, activity, and individual function of IDO1 and its isozymes in gliomas are very limited.

In this study, using paraffin-embedded tissue sections from patients with glioma (75 cases) at different pathologic grades, it was found that the expression of IDO1/TDO rather than IDO2 was positively correlated to the pathologic grades of gliomas. Similar results have been observed for IDO1, IDO2, and TDO mRNA expression in the TCGA database^35^ and IDO1/TDO protein expression of paraffin-embedded tissue sections from glioma patients.^13,31,32,34^ Compared with IDO1/TDO, the function of IDO2 has been less studied. IDO2 plays a nonredundant role in Trp metabolism^36^ and a nonredundant immunosuppressive role different from that of IDO1 and TDO.^37,38^ The function of IDO2 within the central nervous system is still not clear, but it seems to be different from that of IDO1 and TDO.^39^ Therefore, it is not unexpected to find that IDO2 is not correlated with the pathologic grade of gliomas. However, there is a recent report showing that the expression of IDO2 was positively correlated with the grades of glioma.^34^

A previous study using bioinformatical analysis of the Rembrandt or TCGA database showed that high IDO1/TDO mRNA expression was associated with poor prognosis in patients with glioma.^13,35,40^ Herein, for the first time, it was found that glioma patients with positive IDO1/TDO protein expression had shorter overall survival. Previous studies implied the potential involvement of IDO1/TDO in brain edema. For example, 5-aminolevulinic acid, a precursor of IDO1 enzyme synthesis, can induce the formation of brain edema.^41^ In the clinic, dexamethasone, a glucocorticoid that downregulates TDO expression,^42^ is often used as a drug to mitigate patient brain edema.^43–45^ This study revealed a positive correlation between IDO1/TDO expression and AQP4 expression, and a higher incidence rate of brain edema in the IDO1/TDO-positive group than in the IDO1/TDO-negative group.

The concentrations of Trp and Kyn and the Kyn/Trp ratio have been previously used to reflect the total activity of IDO1, IDO2, and TDO.^6^ The Kyn/Trp ratio has been used to evaluate the prognosis of lung cancer, endometrial cancer, and ovarian cancer.^46,47^ This is the first time that the serum Kyn/Trp ratio has been positively correlated with the pathologic grades of gliomas. The reason that the Kyn/Trp ratio in low-grade glioma patients was not higher than that in non-glioma patients might be the limited sample size. IDO2 exhibited much lower catalytic affinity toward Trp than IDO1/TDO, although IDO2 plays a nonredundant role in Trp metabolism.^36^ Given that IDO2 expression was not correlated with the pathologic grades of gliomas, overall survival, or Ki67 index, it can be concluded that the expression and activity of IDO1/TDO rather than IDO2 were positively correlated with the malignancy of gliomas.

TDO is thought to be the main modulator of Trp catabolism, although IDO1 overexpression under a variety of pathological conditions also plays an important role in Trp catabolism.^36,48^ On the basis of the finding that IDO1 and IDO2 did not account for the constitutive Trp catabolism in glioma cells, TDO-derived Kyn activation of AhR has been thought to be a novel mechanism to suppress antitumor immunity and support tumor growth of gliomas.^13^ However, IDO1 possesses the highest affinity for Trp among the three enzymes,^49^ and the contribution of IDO1 to Kyn production cannot be ignored in IDO1-expressing gliomas. Our data showed that the Kyn/Trp ratio and IDO1 expression were positively correlated with the pathologic grades of glioma, which suggested an indispensable function of IDO1 in the pathological process of gliomas. Using a glioma cell line, we found that both IDO1 and TDO contributed to the production of Kyn, which upregulated AhR expression and regulated the migration and invasion of glioma cells. Although the TDO–Kyn–AhR signaling pathway has been reported to promote glioma migration and invasion,^13^ the exact mechanism was not clear. For the first time, we demonstrated that the IDO1/TDO–Kyn–AhR signaling pathway could regulate AQP4, which is involved in the migration and invasion of glioma cells.^18,19^

Some IDO1 inhibitors have been developed and applied in antitumor therapy in preclinical and clinical trials.^50^ Although IDO1 inhibitors have not demonstrated antitumor activity as single agents in orthotopic glioma animals, a unique synergy when combined with radiotherapy, temozolomide, or anti-programmed cell death protein 1 antibody has been noted.^28–30,51–53^ TDO inhibitors have not been tested in glioma animals. In this study, RY103, an IDO1/TDO dual inhibitor, showed excellent therapeutic efficacy via downregulating the IDO1/TDO–Kyn–AhR–AQP4 signal pathway in GL261 orthotopic glioma mice.

**Fig. 8:**
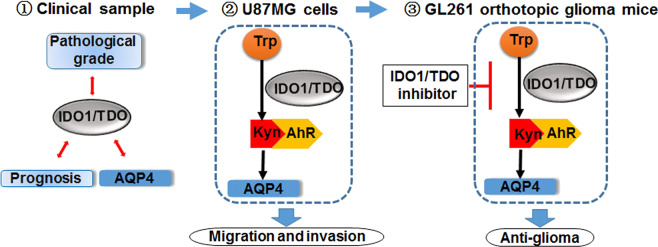
Schematic representation of IDO1 and TDO contributing to the malignancy of gliomas.

In conclusion, using clinical samples, we found that the expression and activity of IDO1/TDO rather than IDO2 contribute to the malignancy of glioma. Using the U87MG glioma cell line, we found that IDO1/TDO accounted for the production of Kyn, which activated AhR to promote cell motility via the Kyn–AhR–AQP4 signaling pathway and modulated the cell area and cytoskeleton. Using GL261 orthotopic glioma mice, the therapeutic effects of the IDO1/TDO dual inhibitor RY103 were confirmed. A summary schematic diagram is shown in Fig. 8. Thus, the IDO1/TDO–Kyn–AhR–AQP4 signaling pathway is a novel mechanism for understanding the malignancy of gliomas. Our study not only provides an improved understanding of the relationship between Trp catabolism and gliomas but also supports anti-glioma drug development targeting IDO1/TDO.

## Materials and methods

### Patient samples

Seventy-five newly diagnosed patients with glioma were classified into grade I, *n* = 6; grade II, *n* = 12; grade III, *n* = 21; grade IV, *n* = 36 according to the World Health Organization (WHO) standard at Xinhua Hospital from January 2015 to December 2017. The clinical characteristics of the 75 patients with glioma are shown in Supplementary Table S1. The detailed information on glioma patients who were followed up is shown in Supplementary Table S2.

In addition, 34 blood samples were also obtained from 16 newly diagnosed patients with glioma (grades I/II, *n* = 5; grades III/IV, *n* = 11) and age- and gender-matched 18 non-glioma patients at Xinhua Hospital. Clinical characteristics of the 34 blood donors are shown in Supplementary Table S4.

Grades I and II were defined as low-grade, and grades III and IV were defined as high-grade gliomas.^54^ The glioma tissue donors and blood donors did not overlap.

The study was approved by the Human Ethics Committee of Fudan University and Xinhua Hospital and was conducted in accordance with the Declaration of Helsinki. Informed consent was obtained from all patients.

### Immunohistochemistry

Paraffin-embedded tissue sections were cut and mounted on slides. After dewaxing and rehydration, antigen retrieval was performed in 10 mmol/L citrate buffer for 30 min at 100 °C. Endogenous peroxidase activity and nonspecific antigens were blocked with 3% hydrogen peroxide and serum. The slides were incubated with anti-IDO1 (1:100, Cell Signaling Technology, USA, no. 86630), anti-IDO2 (1:100, Santa Cruz Biotechnology, USA, no. sc-87164), anti-TDO (1:50, Abcam, UK, no. ab84926), or anti-AQP4 (1:100, Santa Cruz Biotechnology, USA, no. sc-20812) antibody overnight at 4 °C. Slides were then incubated with goat anti-rabbit secondary antibody (1:100, Thermo Fisher Scientific, USA, no. G-21234), developed using 3,3-diaminobenzidine solution and counterstained with hematoxylin.

### Analysis of total activity of IDO1, IDO2, and TDO

The total activity of IDO1, IDO2, and TDO was evaluated by measuring the levels of Trp and Kyn by HPLC.^6^

Blood samples were collected in lithium heparin or ethylene diamine tetraacetic acid vacutainer venous blood collection tubes. The serums were separated from blood samples by centrifugation at 3000 *g* for 15 min and stored at −80 °C.

The serums and cell culture supernatants were treated with 5% perchloric acid and methanol to remove protein, and the supernatants were subjected to HPLC analysis. The analysis was performed on an Agilent 1260 series HPLC system (Agilent Technologies, USA) equipped with a quaternary pump and a UV detector. HPLC analysis of the samples was performed using an Agilent C18 column (5 -μm particle size, L × I.D. 25 cm × 4.6 mm) preceded by a C18 guard column (Dikma, China). The mobile phase (pH 3.6) consisted of 15 mmol/L acetic acid–sodium acetate buffer and acetonitrile at a ratio of 94:6. The detected wavelengths were 280 nm for Trp and 360 nm for Kyn.

### Cell culture and transfection

The glioma cell lines U87MG, U251, A172, and GL261 were purchased from ATCC. The four cell lines were tested and authenticated by short tandem repeat profiling analysis before the first cell experiment, and were used from passage 10 to passage 15. All cells were maintained at 37 °C in an atmosphere of 5% CO_2_ in Dulbecco's modified Eagle medium (Gibco, USA) supplemented with 10% FBS (Gibco, USA) and 1% pen-strep (Gibco, USA). Cells were seeded in a six-well culture dish and transfected at ~70% confluence. Transfection was performed with Lipofectamine 2000 transfection reagent (Invitrogen, USA) according to the manufacturer's instructions. Human IDO1 and TDO cDNA were cloned into the pcDNA3.1( + ) vector, and human AQP4 cDNA was cloned into the pENTER vector. The siRNA targeting human TDO or AQP4 and the negative control siRNA, which had no homology within the human genome, are shown in Supplementary Table S6.

### Cell treatment conditions

U87MG cells were exposed to the following conditions: control, no treatment; IFN-γ, incubated with 100 ng/mL IFN-γ for 24 h; IFN-γ + 1-MT, incubated with 100 ng/mL IFN-γ and 400 μmol/L 1-MT for 24 h; OE-nc, transfected with empty plasmid for 24 h; OE-IDO1, transfected with pcDNA3.1( + )–IDO1 plasmid for 24 h; OE-TDO, transfected with pcDNA3.1( + )–TDO plasmid for 24 h; si-nc, transfected with negative control siRNA for 24 h; si-TDO, transfected with TDO siRNA for 24 h; Kyn, incubated with 150 μmol/L Kyn for 24 h; IFN-γ + 1-MT + Kyn, incubated with 100 ng/mL IFN-γ, 400 μmol/L 1-MT, and 150 μmol/L Kyn for 24 h; si-TDO + Kyn, transfected with TDO siRNA for 24 h, followed by the treatment with 150 μmol/L Kyn for 24 h; SR-1, incubated with 1 μmol/L SR-1 for 24 h; Kyn + SR-1, incubated with 1 μmol/L SR-1 and 150 μmol/L Kyn for 24 h; OE-AQP4, transfected with pENTER-AQP4 plasmid for 24 h; si-AQP4, transfected with AQP4 siRNA for 24 h; si-AQP4 + Kyn, transfected with AQP4 siRNA for 24 h, followed by the treatment with 150 μmol/L Kyn for 24 h. 1-MT (L stereoisomer), IFN-γ, Kyn, and SR-1 were purchased from Sigma-Aldrich (>95% purity).

### RT-PCR and quantitative real-time PCR (qPCR)

The total RNA was extracted from U87MG cells using TRIzol reagent (Invitrogen, USA). RT-PCR was performed to synthesize cDNA using a Premium One-Step RT-PCR kit (Invitrogen, USA). qPCR was performed in triplicate to detect the expression levels of AhR, CYP1A1, CYP1B1, AQP4, and GAPDH using the SYBR Green PCR Master Mix kit (Takara, Japan). GAPDH was used as the internal control. The amplification program consisted of activation at 95 °C for 3 min, followed by 40 amplification cycles consisting of 95 °C for 15 s, 60 °C for 15 s, and elongation at 72 °C for 30 s. The primers used for the qPCR are shown in Supplementary Table S7. Data were analyzed using My IQ software (Bio-Rad, Germany).

### Western blot analysis

U87MG cells and tissues isolated from GL261 orthotopic glioma mice were lysed with RIPA lysis buffer, and supernatants were collected as total protein. Nuclear and cytoplasmic proteins were extracted by a nuclear and cytoplasmic protein extraction kit (Beyotime, China) according to the instructions provided by the manufacturer. Protein concentration was determined with the BCA Protein Assay Reagent (Beyotime, China). Lysates (40 μg) were separated by 10% SDS-PAGE and transferred onto PVDF membranes. After blocking with PBS containing 5% nonfat milk, membranes were incubated with the following primary antibodies: anti-IDO1 (1:1000, Cell Signaling Technology, USA, no. 86630), anti-IDO2 (1:1000, Santa Cruz Biotechnology, USA, no. sc-87164), anti-TDO (1:500, Abcam, UK, no. ab84926), anti-AhR (1:1000, Proteintech, USA, no. 17840-1-AP), anti-AQP4 (1:1000, Santa Cruz Biotechnology, USA, no. sc-20812), anti-GAPDH (1:5000, Proteintech, USA, no. 10494-1-AP), and anti-Histone H3 (1:5000, Abcam, UK, ab1791). After incubation with a HRP-conjugated anti-mouse secondary antibody (1:2000, Thermo Fisher Scientific, USA, no. G-21040) or anti-rabbit secondary antibody (1:2000, Thermo Fisher Scientific, USA, no. G-21234), the proteins were visualized with ECL reagents (Thermo Fisher Scientific, USA), and immunoreactive signals were analyzed by densitometry. The intensity of each target protein band was quantified by densitometry analysis using ImageJ software.

### Cell migration and invasion assays

For migration assays, 5 × 10^4^ cells in 200 μL of serum-free media were seeded in the upper chamber of an insert (8 -μm pore size, Corning, USA). For invasion assays, 2 × 10^5^ cells in 200 μL of serum-free media were seeded into the upper chamber of an insert coated with Matrigel (BD Bioscience, USA). Five-hundred microliters of media containing 10% FBS were added to the lower chamber. After 24 h of incubation, the cells remaining on the top surface of the membrane were gently removed with a cotton swab, while the cells migrating or invading through the membrane were fixed with methanol and stained with 0.1% crystal violet. The entire membrane surface was imaged, and the cells were counted using an upright microscope (Nikon, Japan). Each experiment was performed in triplicate.

### Cell morphology analysis

U87MG cells were seeded in six-well culture dishes. After different treatments, the morphological changes in the cells were observed under an inverted phase-contrast microscope (Olympus, Japan). Images were obtained at ×400 magnification using a digital camera under the same light conditions and exposure times. The areas of 30 cells in several fields were analyzed using ImageJ software, and five independent experiments were performed.

### Fluorescence staining

U87MG cells were grown on glass coverslips and subsequently fixed with 4% paraformaldehyde for 20 min. The fixed cells were then permeabilized with 0.5% Triton X-100 for 30 min. For AhR and AQP4 staining, the fixed cells were incubated with primary antibodies against AhR (1:100, Proteintech, USA, no. 17840-1-AP) and AQP4 (1:100, Santa Cruz Biotechnology, USA, no. sc-20812) at 4 °C overnight after washing with PBS. This procedure was followed by incubation with a secondary antibody (goat anti-rabbit Alexa Fluor 488, 1:1000, Thermo Fisher Scientific, USA, no. A-11034) for 45 min after washing with PBS.

For F-actin staining, the fixed U87MG cells were stained with phalloidin-iFluor 488 (1:500, Thermo Fisher Scientific, USA, no. 21833) for 20 min. The cells were subsequently stained with DAPI (1:1000) for 10 min. Slides mounted with coverslips were viewed under a fluorescence microscope (Olympus, Japan). Images were obtained at ×400 magnification using a digital camera under the same light conditions and exposure times. The fluorescence intensity of F-actin from 30 cells in several fields was analyzed using ImageJ software, and five independent experiments were performed.

### ChIP-qPCR

U87MG cells were cross-linked with 1% formaldehyde for 10 min at room temperature. The fixed cells were lysed and sonicated, the lysates were cleared by centrifugation, and an antibody against AhR was added and incubated overnight under rotation.^55,56^

After reversal of the cross-linking, precipitated DNA was purified with a column and eluted in 20 μL of water. Precipitated DNA fragments were amplified by qPCR. The primers specific for the AQP4 promoter are listed in Supplementary Table S7.

### GL261 orthotopic glioma mouse model construction

Female, 6-week-old, C57BL/6 mice were purchased from SLAC Experimental Animal Center, Shanghai, China. The experimental procedures were approved by the Animal Ethics Committee of Fudan University and performed in compliance with ARRIVE guidelines.

Anesthesia of the mice was achieved with 4% isoflurane (RWD, China) and maintained with 1.75–2.5% isoflurane. The surgical site was shaved and cleaned with iodine. Mice were placed in a stereotactic frame, and the skin that covered the anterior fontanel was cut. A 0.5-mm hand drill was used for drilling holes in skulls. A 2 × 10^5^ GL261 cell suspension in PBS (2 µL) was injected into the right striatum (2 mm lateral, 1 mm anteroposterior from the bregma) at 0.5 µL per min with a needle at a depth of 3 mm. After needle removal, the skin was stapled.

### GL261 orthotopic glioma mouse treatment

Five days after the surgery, the GL261 orthotopic glioma mice were divided randomly into a control group and an RY103 group. Drug administration began on the 6th day after operation, with the control group receiving 10% hydroxypropyl-β-cyclodextrin (HPBCD) in normal saline, by intraperitoneal injection (i.p.) every 36 h. The RY103 group received 6 mg/kg RY103 in 10% HPBCD, i.p. per 36 h. Mice were killed after 21 days of therapy. The blood and brain were collected. RY103 was developed by our lab.

The survival times of the control group and RY103 group were compared. Mice were killed when they were moribund (the weight of mice dropped by 15%).

### MRI

GL261 orthotopic glioma mice were first anesthetized with 1.5–2.5% isoflurane and 0.8 L/min O_2_ and placed in a 72-mm quadrature volume coil. The tumor volume was assessed by MRI using 7.0-T small-animal magnetic resonance scanners (Germany, Brooke). Tumor volume was calculated from T2WI high-resolution horizontal images using Paravision 5.1 software (Bruker BioSpin, Ettlingen, Germany).

### Hematoxylin and eosin staining

For histology analysis, the total brains of GL261 orthotopic glioma mice were fixed in 4% paraformaldehyde. Coronal brain slices (5 μm) were stained with hematoxylin and eosin (Hematoxylin and Eosin Staining Kit, Beyotime, China).

### Statistical analysis

Stained slides of glioma tissue samples were reviewed and scored independently by two pathologists blinded to the clinical parameters. The histochemistry score (H score) ranged from 0 to 300 and was calculated as the percentage of weakly stained cells plus the percentage of moderately stained cells multiplied by two plus the percentage of strongly stained cells multiplied by three.^13^ The final H score was the average of H scores obtained by the two pathologists.

We designated the different glioma tissue samples as follows. We defined the IDO1- or TDO-negative (IDO1− or TDO−) samples, those in which < 1% of tumor cells expressed IDO1 or TDO, and IDO1- or TDO-positive (IDO1 + or TDO + ) samples as those in which ≥ 1% of the tumor cells expressed IDO1 or TDO. We defined the IDO1 or TDO-high samples (IDO1 or TDO high) as those whose IDO1 or TDO H score was higher than the mean H score of the IDO1 + or TDO + samples. We also defined the IDO1 or TDO-low samples (IDO1 or TDO low) as those whose IDO1 or TDO H score was lower than the mean H score of the IDO1 + or TDO + samples. The grouping of the glioma tissue samples based on the expression of IDO1/TDO is shown in Supplementary Table S3.

We defined the Ki67-high samples (Ki67 high) as those whose Ki67 index was higher than the mean Ki67 index of all samples. We also defined the Ki67-low samples (Ki67 low) as those whose Ki67 index was lower than the mean Ki67 index of all samples.

Survival curves were calculated according to the Kaplan–Meier method. Hazard ratios (HR) were used to examine the prognostic impact of IDO1, IDO2, TDO, and Ki67 on overall survival in patients with glioma. The *p*-values were obtained by log-rank statistical analysis. Correlations between Ki67 and IDO1, IDO2, or TDO were analyzed by Spearman rank correlation (SPSS, IBM, USA). Fisher’s exact tests were used to compare categorical variables across groups. Cell area and fluorescence intensity were analyzed using ImageJ software. Experimental assessments were performed by investigators who were blinded to group assignments. All experiments were repeated at least three times. Data are expressed as the means ± standard error of the mean (SEM). One-way analysis of variance (ANOVA) followed by Dunnett’s post hoc test was used to compare several treatment groups with one control group. Student’s *t* test was used to determine the difference between two groups. Significance values were set at *p < 0.05, **p < 0.01, ***p < 0.001, and ****p < 0.0001.

The “*n*” in the figure legends indicates the number of patients, mice, or independent cell culture preparations.

## Supplementary information

Supplementary Materials
