# Correction: Both IDO1 and TDO contribute to the malignancy of gliomas via the Kyn–AhR–AQP4 signaling pathway

**DOI:** 10.1038/s41392-021-00808-9

**Published:** 2021-11-08

**Authors:** Lisha Du, Zikang Xing, Bangbao Tao, Tianqi Li, Dan Yang, Weirui Li, Yuanting Zheng, Chunxiang Kuang, Qing Yang

**Affiliations:** 1 State Key Laboratory of Genetic Engineering, School of Life Sciences, Fudan University, Songhu Road 2005, Shanghai, 200438 China; 2 Department of Neurosurgery, Xinhua Hospital, Shanghai Jiaotong University, School of Medicine, Kongjiang Road 1665, Shanghai, 200092 China; 3 Department of Chemistry, Tongji University, Siping Road 1239, Shanghai, 200092 China; 4 Institute of Science and Technology for Brain-Inspired Intelligence, Fudan University, Handan Road 220, Shanghai, 200433 China

**Keywords:** CNS cancer, Diseases of the nervous system

Correction to: *Signal Transduction and Targeted Therapy* 10.1038/s41392-019-0103-4, published online 21 Feb 2020

In the process of collating the raw data, the authors noticed the inadvertent mistakes in Fig. 4h & 6g that need to be corrected. The correct data are provided as follows. The key findings of the article are not affected by the corrections. We apologize for the inadvertent mistakes.
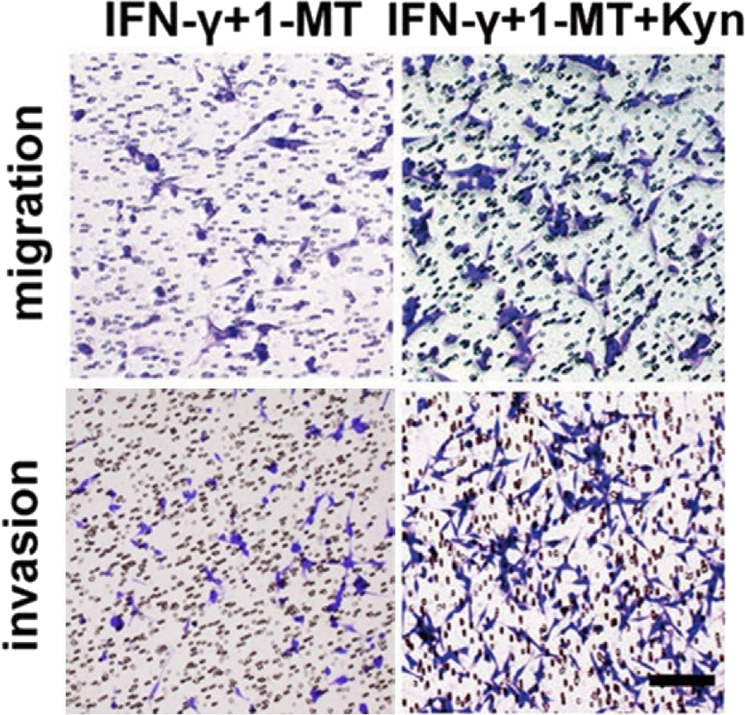


**Fig. 4**h Migration and invasion assays of U87MG cells treated with IFN-γ + 1-MT or IFN-γ + 1-MT + Kyn.

The authors mistakenly placed the wrong representative image showing the invasion ability of U87MG cells in the IFN-γ + 1-MT group. The correct version of Fig. 4h is shown above.
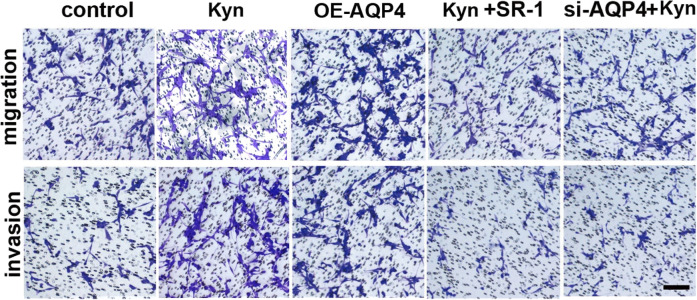


**Fig. 6**g Migration and invasion assays of U87MG cells under different conditions (magnification, ×200; scale bar, 100 μm).

The authors mistakenly placed the wrong representative images showing the migration and invasion abilities of U87MG cells in the Kyn group. The correct version of Fig. 6g is shown above.

