# Supplementary material for: Both IDO1 and TDO contribute to the malignancy of gliomas via the Kyn–AhR–AQP4 signaling pathway
Source: Signal Transduct Target Ther. 2020 Feb 21;5:10. doi: 10.1038/s41392-019-0103-4 (PMC7033114; doi:10.1038/s41392-019-0103-4)
Supplement: Supplementary file 1 — Supplementary Materials [file 41392_2019_103_MOESM1_ESM.docx]

Supplementary Materials for

Both IDO1 and TDO contribute to the malignancy of gliomas via Kyn-AhR-AQP4 signal pathway

Lisha Du^1^, Zikang Xing^1^, Bangbao Tao^3^, Tianqi Li^1^, Dan Yang^1^, Weirui Li^1^, Yuanting Zheng^1^, Chunxiang Kuang^4^, Qing Yang^1, 2,^ *

Correspondence to: [yangqing68@fudan.edu.cn](mailto:yangqing68@fudan.edu.cn)

**This PDF file includes:**

Figures. S1 to S6

Tables S1 to S7


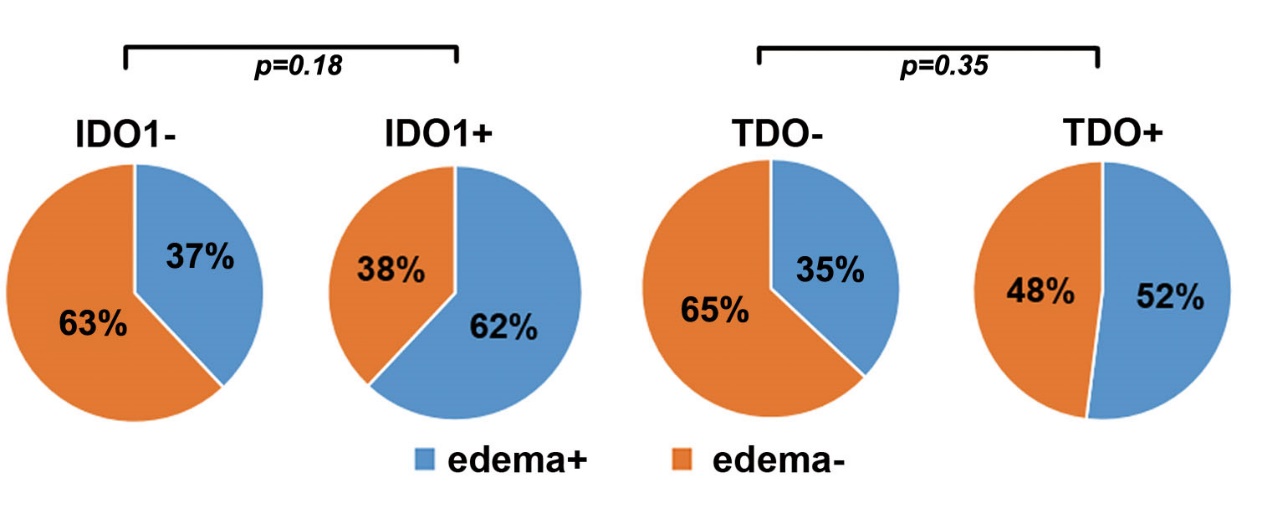
Figure. S1.

Figure S1. The relationship between IDO1/TDO expression and brain edema in glioma patients. The incidence rates of edema in IDO1 negative (IDO1-) (n=30), IDO1 positive (IDO1+) (n=13), TDO negative (TDO-) (n=20) and TDO positive (TDO+) (n=23) groups were respectively calculated. Statistical significance was determined by Fisher’s exact tests.

Figure. S2.


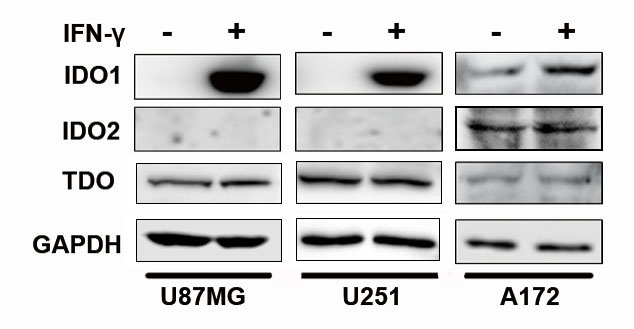


Figure S2. Western blot analysis of the expressions of IDO1, IDO2 and TDO in different human glioma cells in the absence or presence of IFN-γ (100 ng/mL). n=3 per group.

Figure. S3.

**
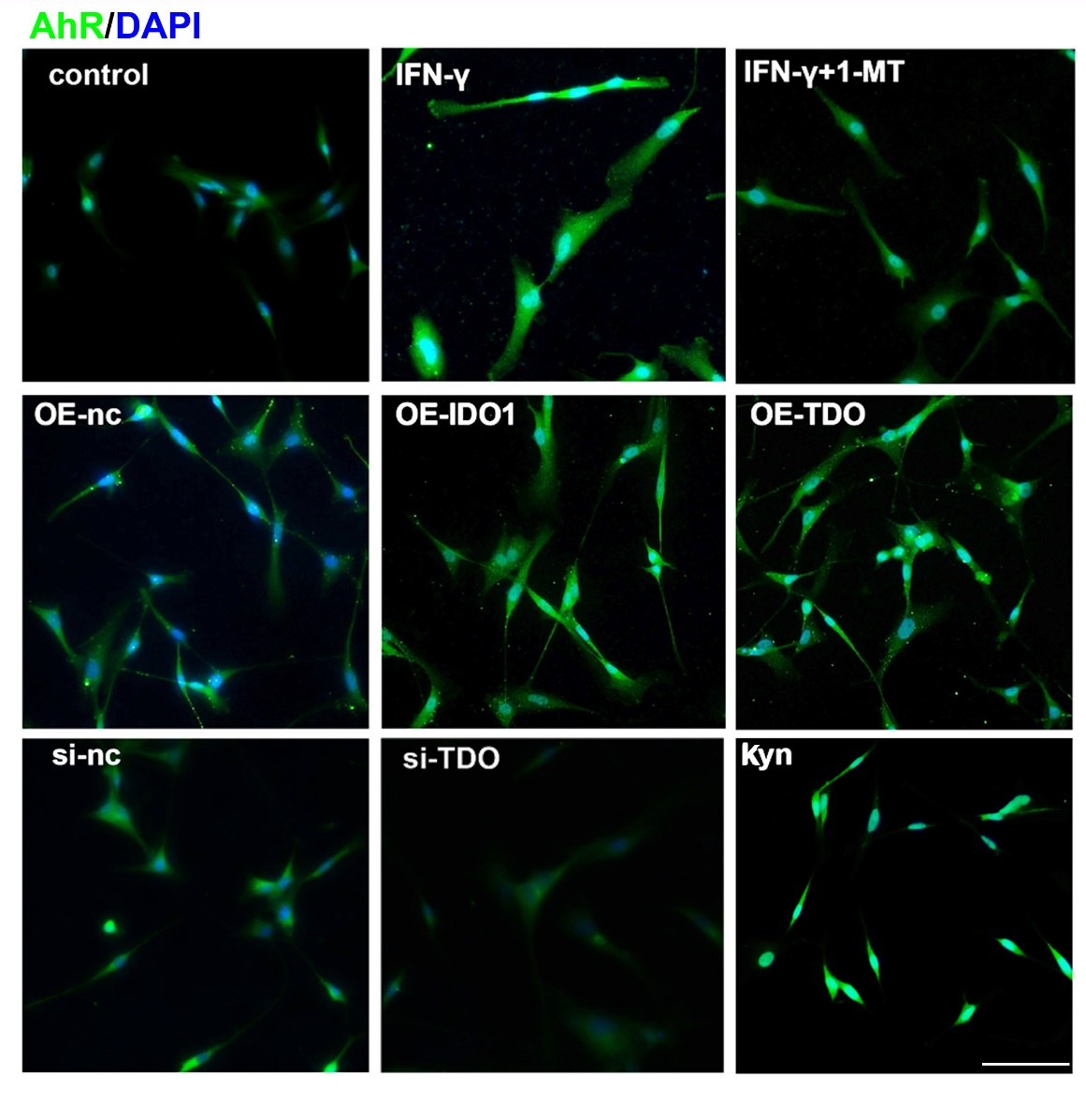
**

Figure S3. Immunofluorescence analysis of the effects of IDO1, TDO and Kyn on AhR expression (green) in U87MG cells. The designation of different treatments was described in the Materials and Methods. n=5 per group. DAPI (blue) was used for nuclear staining (Magnification, 400×; scale bar, 50 μm).

Figure. S4.

**
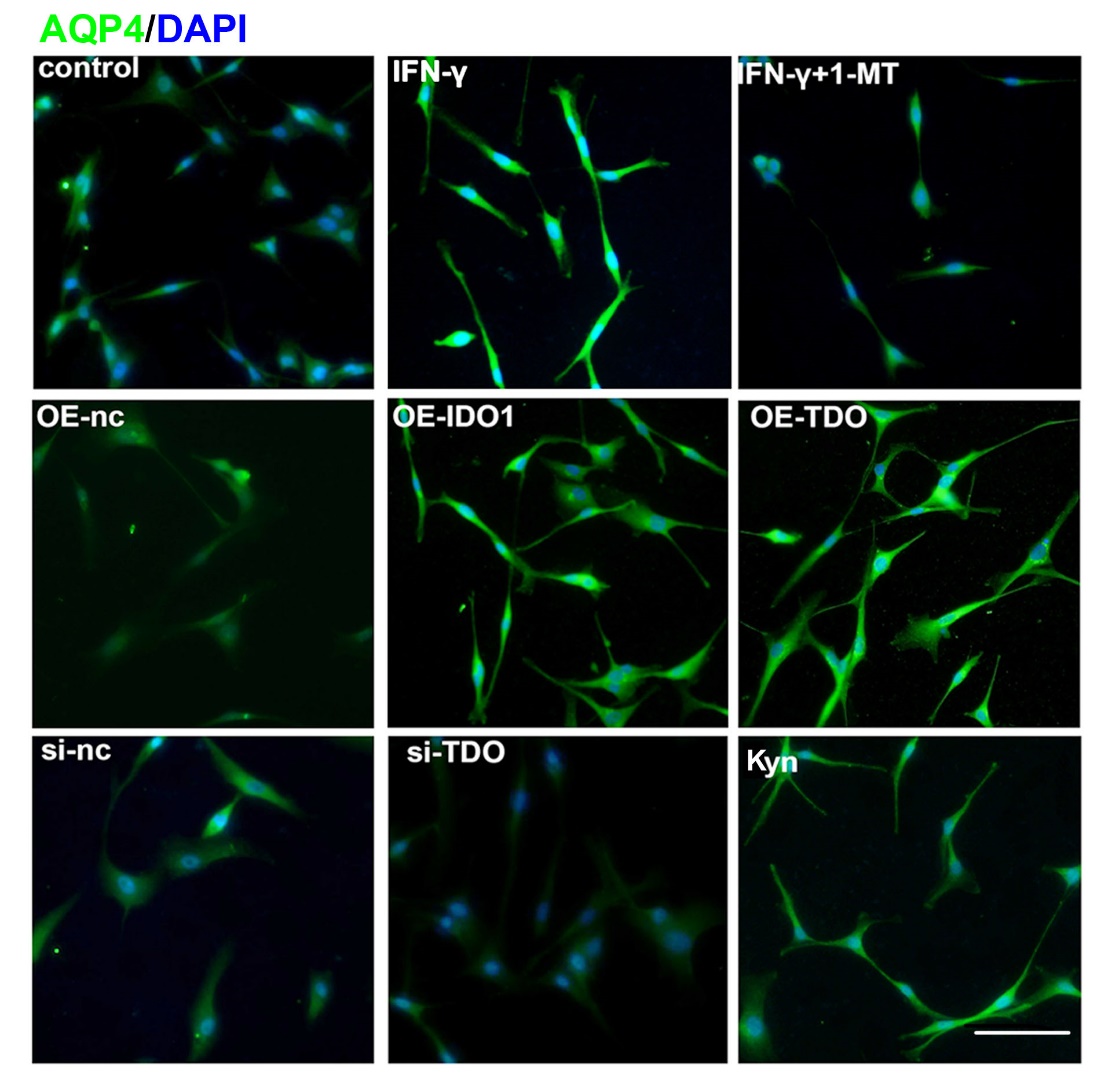
**

Figure S4. Immunofluorescence analysis of the effects of IDO1, TDO and Kyn on AQP4 expression (green) in U87MG cells. The designation of different treatments was described in the Materials and Methods. n=5 per group. DAPI (blue) was used for nuclear staining (Magnification, 400×; scale bar, 50 μm).

Figure. S5.

**
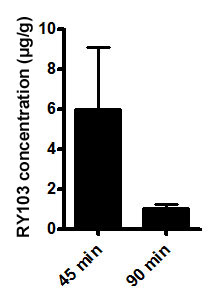
**

Figure S5. RY103 was able to penetrate the blood brain barrier in mice. The concentration of RY103 in C57BL/6 mice brain was analyzed by LC-MS. Mice were administrated with a single dose of RY103 (6 mg/kg in 10% HPBCD, i.p.), and were sacrificed to isolate brain tissues at 45 min and 90 min after the administration (n=3 per group).

Figure. S6.

**
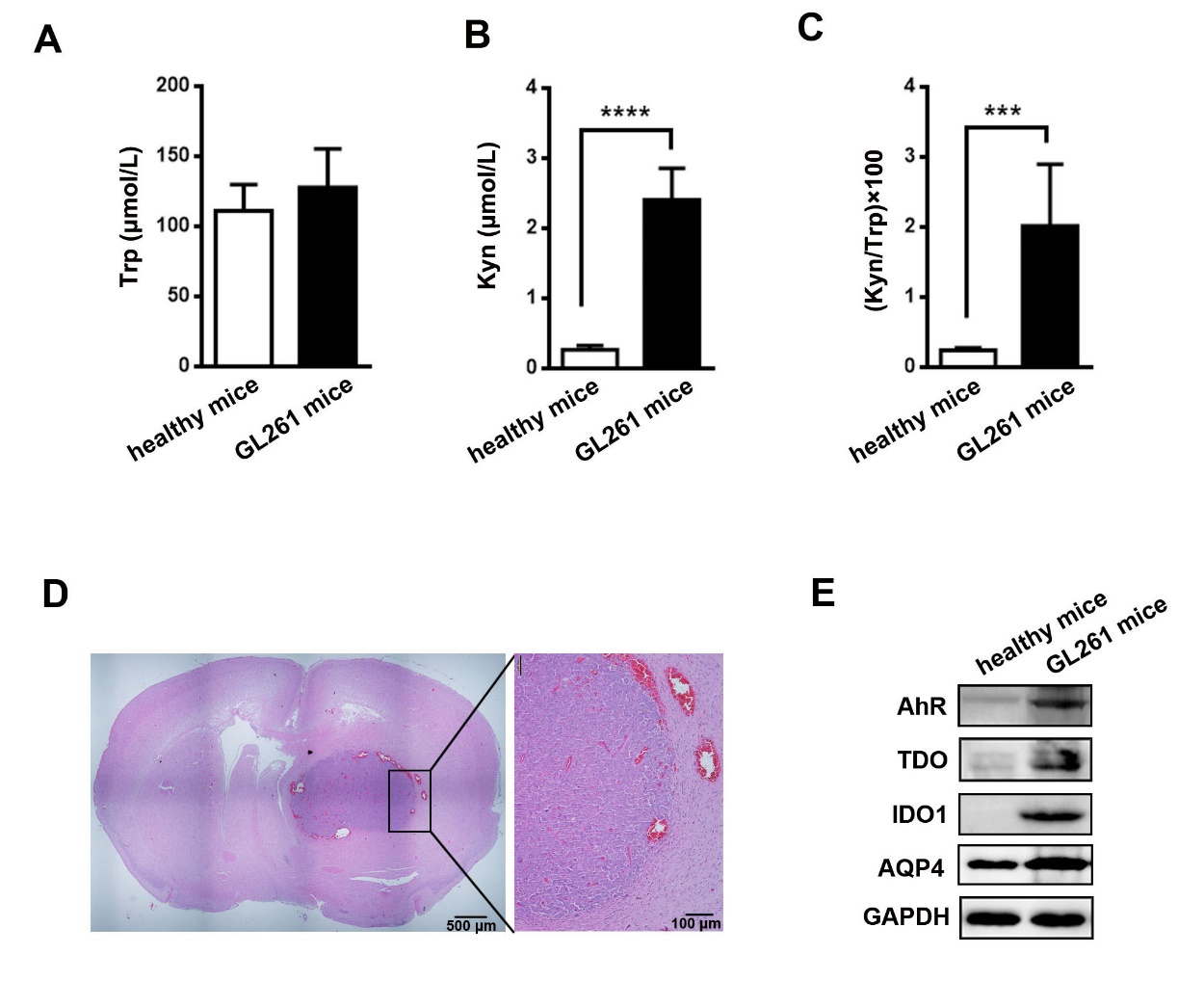
**

Figure S6. IDO1/TDO-Kyn-AhR-AQP4 pathway was upregulated in GL261 orthotopic glioma mice. (A-C) HPLC analysis of Trp, Kyn and the Kyn/Trp ratios in healthy mice (n=6) and GL261 orthotopic glioma mice (GL261 mice) (n=7). (D) Hematoxylin and eosin staining of representative coronal brain sections from GL261 mice, left (40×, scale bar 500 μm), right (200×, scale bar 100 μm). (E) Western blot analysis of the expressions of IDO1, TDO, AhR and AQP4 in both the glioma tissues from GL261 mice and the corresponding brain tissues from healthy mice (n=3 per group). Statistical significance was determined by Student's t test (A-C). Data were presented as the mean ± SEM. ****p* < 0.001; *****p* < 0.0001.

Table S1.

**Table S1.** Basic clinical characteristics of 75 glioma

tissue donors.

| **Variable** | **Number** |
| --- | --- |
| Gender |  |
| Male | 44 |
| Female | 31 |
| Age at diagnosis, year |  |
| < 50 | 46 |
| ≥ 50 | 29 |
| Pathologic grades |  |
| I | 6 |
| II | 12 |
| III | 21 |
| IV | 36 |
| Ki67 |  |
| < mean | 32 |
| ≥ mean | 31 |
| No information | 12 |
| Brain edema |  |
| Yes | 20 |
| No | 23 |
| No information | 32 |
| Survival information |  |
| Live | 26 |
| Death | 11 |
| Loss of follow up | 6 |
| No information | 32 |

The mean of Ki67 was 30%.

Table S2.

**Table S2.** Detailed information of glioma patients who were followed up.

| Pathologic grades | I (n=6) | II (n=4) | III (n=2) | IV (n=31) | Total (n=43) |
| --- | --- | --- | --- | --- | --- |
| Ki67 |  |  |  |  |  |
| ≥mean | 0 | 0 | 1 | 22 | 23 |
| < mean | 4 | 4 | 1 | 8 | 17 |
| No information | 2 | 0 | 0 | 1 | 3 |
| Survival information |  |  |  |  |  |
| Live | 5 | 3 | 0 | 18 | 26 |
| Death | 0 | 1 | 0 | 10 | 11 |
| Loss of follow up | 1 | 0 | 2 | 3 | 6 |
| IDO1+ | 2 | 1 | 1 | 9 | 13 |
| IDO1- | 4 | 3 | 1 | 22 | 30 |
| IDO2+ | 0 | 0 | 1 | 11 | 12 |
| IDO2- | 6 | 4 | 1 | 20 | 31 |
| TDO+ | 2 | 2 | 2 | 17 | 23 |
| TDO- | 4 | 2 | 0 | 14 | 20 |

IDO1+: IDO1 positive; IDO1-: IDO1 negative; IDO2+: IDO2 positive; IDO2-: IDO2 negative;

TDO+: TDO positive; TDO-: TDO negative.

Table S3.

**Table S3.** The grouping of the glioma tissue samples

based on the expression of IDO1/TDO.

| **Variable** | **Number** |
| --- | --- |
| IDO1+ | 17 |
| IDO1 high (≥mean) | 7 |
| IDO1 low (<mean) | 10 |
| TDO+ | 41 |
| TDO high (≥mean) | 17 |
| TDO low (<mean) | 24 |

The mean of IDO1 H score was 94.18. The mean of TDO H score

was 93.29.

Table S4.

**Table S4.** Basic clinical characteristics of 34 blood donors.

|  | **Variable** | **Number** |
| --- | --- | --- |
| Glioma patients  n=16 | Gender |  |
|  | Male | 9 |
|  | Female | 7 |
|  | Age at diagnosis, year |  |
|  | < 50 | 5 |
|  | ≥ 50 | 11 |
|  | Pathologic grades |  |
|  | Low grade (I/II) | 5 |
|  | High grade (III/IV) | 11 |
| Non-glioma patients  n=18 | Gender |  |
|  | Male | 9 |
|  | Female | 9 |
|  | Age at diagnosis, year |  |
|  | < 50 | 8 |
|  | ≥ 50 | 10 |

Table S5.

**Table S5.** The incidence rate of brain edema in glioma patients with positive

expression of IDO1, IDO2 and TDO.

| **Group** | **Total (n)** | **Brain edema (n)** | **Incidence rate** |
| --- | --- | --- | --- |
| IDO1+ | 13 | 8 | 62% |
| IDO1- | 30 | 11 | 37% |
| TDO+ | 23 | 12 | 52% |
| TDO- | 20 | 7 | 35% |

IDO1+: IDO1 positive; IDO1-: IDO1 negative; TDO+: TDO positive; TDO-: TDO negative

Table S6.

**Table S6.** siRNA sequences.

| **siRNA** | | **Sequences (5’-3’)** |
| --- | --- | --- |
| si-nc | sense | UUCUCCGAACGUGUCACGUTT |
|  | antisense | ACGUGACACGUUCGGAGAATT |
| si-TDO | sense | GGAGUUGGAUUCUGUUCGA |
|  | antisense | UCGAACAGAAUCCAACUCC |
| si-AQP4 | sense | GUGGCCUUUAUGAGUAUGUTT |
|  | antisense | ACAUACUCAUAAAGGCCACTT |

Table S7.

**Table S7.** The sequences of primers used for qPCR analysis of gene expression.

| **Primer** | **Sequences (5’-3’)** |
| --- | --- |
| AQP4-P1-forward | GCAGTCCGCCTGGATCTGAATC |
| AQP4-P1-reverse | CTTAGCTTCCTGCTCCTTAGTG |
| AQP4-P2-forward | CGGTGGTGAACTCTATTTCTGG |
| AQP4-P2-reverse | CTTTTCATGAATCTCAGCATCA |
| AQP4-P3-forward | GAAAATAGTATGGCATAGTAG |
| AQP4-P3-reverse | GGTCTTTATGCCAGACCACCTT |
| AQP4-P4-forward | CACCTCCTGGTGGTCCACCAC |
| AQP4-P4-reverse | TGTGATACTGTCAGCTCTATGC |
| AQP4-P5-forward | GCTACTCTGCTGAGAGTACTA |
| AQP4-P5-reverse | CTCGGCAGTTGCAGTTACCA |
| AQP4-P6-forward | CTTTCTTGTCATZCTTCATTCTG |
| AQP4-P6-reverse | TGTAATGAACATCTATGAACC |
| GAPDH-forward | CAATGACCCCTTCATTGACC |
| GAPDH-reverse | TGGAAGATGGTGATGGGATT |
| AhR-forward | ATTGTGCCGAGTCCCATATC |
| AhR-reverse | AAGCAGGCGTGCATTAGACT |
| CYP1A1-forward | CTTGGACCTCTTTGGAGCT |
| CYP1A1-reverse | GACCTGCCAATCACTGTG |
| CYP1B1-forward | GACGCCTTTATCCTCTCTGCG |
| CYP1B1-reverse | ACGACCTGATCCAATTCTGCC |
| AQP4-forward | AGTGTTATGCCTGGGAGTGC |
| AQP4-reverse | CGGTGCAAACCATCTTTGGG |
